# Carriage Prevalence of Extended-Spectrum β-Lactamase Producing Enterobacterales in Outpatients Attending Community Health Centers in Blantyre, Malawi

**DOI:** 10.3390/tropicalmed6040179

**Published:** 2021-09-29

**Authors:** Onduru Gervas Onduru, Rajhab Sawasawa Mkakosya, Susan Fred Rumisha, Said Aboud

**Affiliations:** 1 The Africa Center of Excellence in Public Health and Herbal Medicine (ACEPHEM), Kamuzu University of Health Sciences, Blantyre Private Bag 360, Malawi; 2 Department of Pathology, Kamuzu University of Health Sciences, Blantyre Private Bag 360, Malawi; rmkakosya@medcol.mw; 3 Directorate of Information Technology and Communication, National Institute for Medical Research, P.O. Box 9653 Dar es Salaam, Tanzania; siaeli@gmail.com; 4 Malaria Atlas Project, Geospatial Health and Development, Telethon Kids Institute, Perth, WA 6009, Australia; 5 Department of Microbiology and Immunology, Muhimbili University of Health and Allied Sciences, P.O. Box 65001 Dar es Salaam, Tanzania; aboudsaid@yahoo.com

**Keywords:** extended-spectrum β-lactamases, ESBLs, Enterobacterales, community, Malawi

## Abstract

Antimicrobial resistance due to extended-spectrum β-lactamase (ESBL) production by Enterobacterales is a global health problem contributing to increased morbidity and mortality, particularly in resource-constrained countries. We aimed to determine the prevalence of extended-spectrum β-lactamase-producing Enterobacterales (ESBL-E) in community patients in Blantyre, Malawi. Clinical samples were collected from 300 patients and screened for ESBL-E using a CHROMagar^TM^ ESBL medium. Confirmation of ESBL production was done by a combination disk test (CDT). The prevalence of community-acquired ESBL-E was 16.67% (50/300, 95% CI = 12.43–20.91%). The most common ESBL-E species isolated was *Escherichia coli* (66%). All ESBL-E isolates were resistant to Trimethoprim-Sulfamethoxazole except for 2% of *E. coli*. Besides this, all ESBL-E were susceptible to Imipenem and only 4% were resistant to Meropenem. No patients with a positive ESBL-E phenotype had a history of hospital admission in the last three months, and the carriage of ESBL-E was neither associated with the demographic nor the clinical characteristics of participants. Our findings reveal a low presence of ESBL-E phenotypes in community patients. The low prevalence of ESBL-E in the community settings of Blantyre can be maintained if strong infection and antimicrobial use-control strategies are implemented.

## 1. Introduction

Extended-spectrum β-lactamase-producing Enterobacterales (ESBL-E) were exclusively observed in hospital settings in the early years after the introduction of Cephalosporins in 1990s [1,2]. They continue to be a major global health challenge in both clinical and infection prevention control. Currently, a global problem of antimicrobial resistance (AMR) resulting from the production of ESBL enzymes by Enterobacterales has increased to an alarming magnitude and is among the leading threats to human health [3]. The most affected are the developing countries with limited resources to implement control measures to curb AMR [4]. Increased spread of ESBL-E is not only occurring in hospital settings but also in the communities. The prevalence of community-acquired ESBL-E worldwide remained (below 10%) before 2008 but has rapidly exploded to over 60% in recent years, causing a major challenge for antibiotic therapy to combat both nosocomial and community-acquired infections [5,6].

In low-income countries, lack of enforcement of regulations and guidelines controlling the sale and irrational use of antibiotics in both animal and humans contributed to the emergence and widespread of resistant bacteria [7]. The problem with AMR, as a result of an inability to treat bacteria that are resistant to locally available antimicrobial agents, is that it is implicated in longer hospitalization times and the need for more expensive drugs, which are often not readily available in resource-constrained countries like Malawi [8,9].

The few available studies conducted in Malawi to account for the presence of ESBL-E in humans were based on observational study and surveillance data from hospital settings [10,11,12]. In 2005, the prevalence of ESBL-E was 0.7%, reported after a baseline review of the ESBL problem and characterization of ESBL enzymes from clinical isolates following the death of patients attributed to multidrug-resistant *Klebsiella pneumoniae* infection [11]. Twelve years later (2017), a study describing longitudinal trends in AMR at a teaching hospital in Blantyre, Malawi that focused on the prevalence of ESBL resistance among gram-negative bacteria causing blood-stream infections found that the proportion of ESBL-E had risen in *Escherichia coli* from 0.7 to 30.3%, 11.8 to 90.5% in *Klebsiella* spp. and 30.4 to 71.9% in other Enterobacterales [12].

Information on the current status of the ESBL-E burden in the community settings in Malawi—and Blantyre, in particular—is lacking. However, as an evidence of the level of ESBL-E carriage in the communities in Blantyre, ESBL-producing *E. coli* has been recovered from a stool of a patient with neither history of hospital admission nor having traveled in the previous six months [13]. Therefore, the current study was conducted to provide information on the baseline prevalence and antimicrobial susceptibility pattern of ESBL-E in community settings in Blantyre, Malawi.

## 2. Materials and Methods

### 2.1. Study Design, Duration and Location

**Figure 1:**
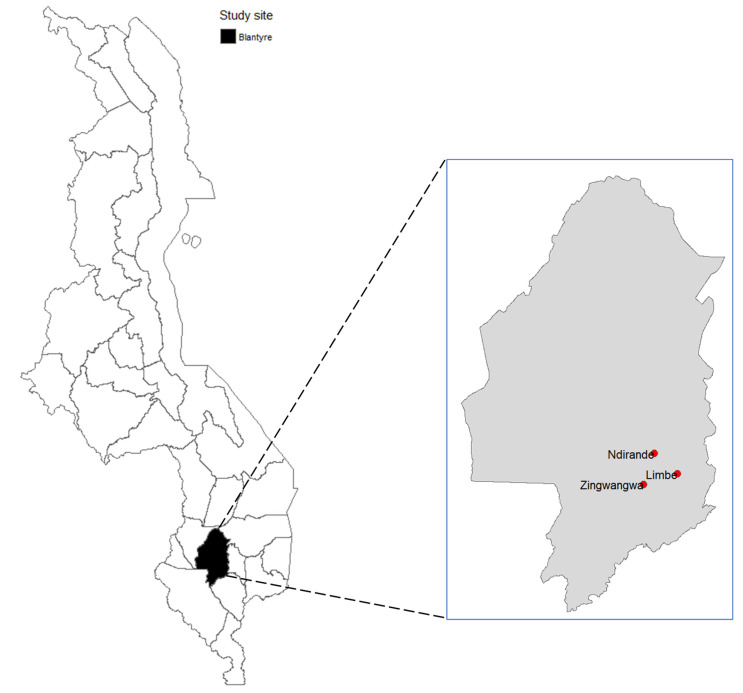
Map of Malawi (**left**) showing location of Blantyre district, and map of Blantyre (**right**) showing locations of health centers where data were collected for this study.

This was a cross-sectional study that was carried out between March and September 2020 at three conveniently selected outpatient health centers representing community settings in Blantyre, Malawi (Figure 1). Outpatient health centers were chosen as points of data collection due to the convenience of sample collection, storage and transportation. *These health centers were Limbe, Zingwangwa and Ndirande*. These health centers are the main public healthcare facilities providing outpatient services to a population of over 800,264 in urban areas of Blantyre district.

### 2.2. Study Population, Sample Size and Sampling

The study participants comprised 300 adult outpatients from three main public health centers chosen to represent community settings in Blantyre. The sample size was calculated using a formula described by Lwanga and Lemeshow [14] based on the 61.9% prevalence of ESBL-E previously reported in Blantyre [12]. All adult patients (≥18 years old) who presented on the day of data collection had an equal chance of being included in the study. Patients who consented to participate were randomly recruited (first come, first served) to join the study regardless of their reason to seek medical care at the health center.

### 2.3. Specimen Collection

Clinical specimens (urine and rectal swabs) were collected and handled following a standard protocol by registered and experienced health personnel working at each health center in accordance with ethical principles for medical research involving human subjects. Only one type of clinical specimen was collected from each participant. Urine samples were exclusively collected from outpatients who presented with UTI-related complaints using a sterile urine-cup, and for all other patients, rectal swabs were instead collected using Amies flocked swabs (COPAN, Brescia-Italia). The collected urine and swabs were transported to the Microbiology Laboratory, College of Medicine, University of Malawi (Currently Kamuzu University of Health Sciences) for culture of the ESBL-E.

### 2.4. Screening for ESBL-E

The culture of ESBL-E was performed on a CHROMagar^TM^ ESBL medium composed of an ESBL supplement containing a selective mixture of antibiotics enabling the selective growth of ESBL-E while inhibiting the growth of non-ESBL-E (CHROMagar, Paris, France). Samples were processed by direct inoculation onto CHROMagar^TM^ ESBL plates using streaking and spreading techniques followed by cover bottom side incubation in aerobic conditions at 37 °C for 18–24 h. Following incubation, significant growth of ESBL-E and appearance of colonies were observed.

### 2.5. Identification of Enterobacterales Species

Presumptive identification of common ESBL-E isolates was done based on the colony color characteristics of the bacterial growth on the CHROMagar^TM^ plates according to the manufacturer’s instructions; i.e., ESBL-producing *Escherichia coli* was identified by a dark pink color, ESBL-producing KEC (*Klebsiella*, *Enterobacter*, *Citrobacter*) by metallic blue +/− reddish halo), ESBL-producing *Proteus* by a brown halo, ESBL = producing *Acinetobacter* by a cream color and ESBL-producing *Pseudomonas* as translucent growth (+/− natural).

The identities of the isolates were subsequently confirmed using commercially acquired biochemical substrate strips (Microbact™ gram-negative identification system, Oxoid, GNB 12A). The standardized micro-substrate strips (Microbact^TM^) were inoculated according to the manufacturer’s instructions for the identification of Enterobacterales. The biochemical tests used were Lysine, Ornithine, H_2_S, Glucose, Mannitol, Xylose, ONPG, Indole, Urease, VP, Citrate and TDA. The interpretation to identify the isolates was done using the Microbact™ computer-aided identification package (Oxoid) in combination with Cowan and Steel’s Manual for the Identification of Medical Bacteria [15].

### 2.6. Phenotypic Confirmation of ESBL Production by Enterobacterales

Phenotypic confirmation of ESBL production by the isolates was done using a combination disk test method (CDT) by comparing the inhibition zone diameter around a cephalosporin disk to that of the same cephalosporin plus clavulanate, following the recommendations of the Clinical and Laboratory Standards Institute (CLSI, 2020) [16]. In the current study, we used MAST combination disks (MAST D52C ESBL; Mast Diagnostics, Merseyside, UK) to phenotypically confirm ESBL production in Enterobacterales. Both cefotaxime (CTX-30 μg) and ceftazidime (CAZ-30 μg) antibiotic disks with and without clavulanic acid (CA-10 μg) were used concurrently based on comparison of the inhibition zones of cefotaxime and ceftazidime disks with and without clavulanic acid. An increase in the inhibition zone diameter of ≥5 mm or a zone expansion of 50%—i.e., corresponding to a two-fold dilution between the inhibition zone of a single disk and in combination with clavulanic acid—was indicative of ESBL production as previously described [17].

### 2.7. Antimicrobial Susceptibility Testing (AST)

The standard Kirby–Bauer disk diffusion method was used to determine the antimicrobial susceptibility of the isolates on a Mueller–Hinton agar media (Merck, Darmstadt, Germany). A panel of 11 different commercially acquired antibiotic disks (Mast, Bootle, Merseyside, UK) was used, including Amikacin (30 μg), Amoxicillin (10 μg), Cefepime (30 μg), Ceftriaxone (30 μg), Ciprofloxacin (5 μg), Doxycycline (30 μg), Gentamicin (10 μg), Trimethoprim-Sulfamethoxazole (2/25 μg), Meropenem (10 μg), Imipenem (10 μg) and Nitrofurantoin (300 μg). For each antibiotic disk, the diameter of the inhibition zone was measured and interpreted according to the Clinical and Laboratory Standards Institute (CLSI, 2020) guidelines [16].

### 2.8. Quality Control

ESBL-producing *Klebsiella pneumonia* (ATCC 700603) and non-ESBL-producing *E. coli* (ATCC 25922) were used as positive and negative controls, respectively.

### 2.9. Statistical Analysis

Data obtained were cleaned and transferred to STATA version 12.0 (Stata Corp LP, College Station, TX, USA) for statistical analysis. Descriptive summary statistics were generated as frequencies and proportions, presented in tables and bar charts. Chi squared/Fisher’s exact tests were used to compare dichotomous variables as appropriate. Univariate association between ESBL-E positivity (outcome) and independent variables was determined by logistic regression analysis. When fitting the model, participants who had separated, divorced, been widowed or who held a single marital status were combined to obtain a single variable (unmarried); these participants were then compared with the married and/or cohabiting participants. The results were presented based on the odds ratio and 95% confidence interval. A *p*-value ≤ 0.05 was regarded as statistically significant.

## 3. Results

### 3.1. Social Demographic and Clinical Characteristics of the Study Population

A total of 300 adult patients attending outpatient clinics in Blantyre district were enrolled in the study. The median age of the participants was 29.5 years (IQR = 23–38; range, 18–75 years). Participant’s male to female sex ratio was 1:1.2. The majority of participants (61.33%) were either married or cohabiting, 46% were unemployed, 44.33% had primary education and 41.67% were between the ages of 18–27 years. In the past three months, more than 97% of participants had no history of admission, 90.33% had no history of surgery and 78% had not used antibiotics (Table 1).

### 3.2. Prevalence of ESBL-E in Community Patients in Blantyre

Of the 300 community patient samples (199 rectal swabs and 101 urine-cups) screened for potential ESBL-E phenotypes, the rate of confirmed ESBL-E carriage was 16.67% (50/300, 95% CI = 12.43–20.91%; 14% rectal carriage and 2.67% in urine).

Of the 50 ESBL-E isolates recovered from adult outpatients in Blantyre, the majority were from rectal swabs (42/50, 84%). The most common ESBL-E species isolated were *Escherichia coli* (33/50, 66%), followed by *Klebsiella* spp. (4/50, 8%) and *Yersinia enterocolitica* (3/50, 6%) (Table 2). The community prevalence of ESBL-E was higher in males (28/50, 56%) compared to females (22/50, 44%); however, this difference was not statistically significant (X^2^ = 2.58, *p* = 0.11). We found a high prevalence of ESBL-E in the married and/or cohabiting (28/50, 56%), unemployed (24/50, 48%) and those with primary education (23/50, 46%). Community patients who lacked a history of surgery or antibiotic use in the past three months (44/50, 88%, X^2^ = 0.37, *p* = 0.54 and 40/50, 80%, X^2^ = 0.14, *p* = 0.71, respectively) had a high rate of ESBL-E carriage compared to those with contrasting characteristics. We observed that no community patients with ESBL-E carriage had a history of hospital admission in the past three months (Table 1).

### 3.3. Antimicrobial Resistance Profiles of Commonly Isolated ESBL-E

**Figure 2:**
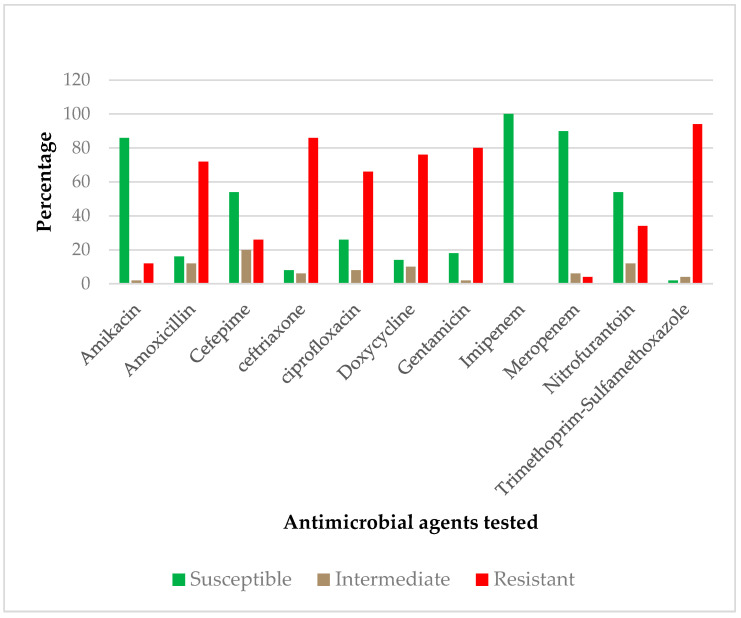
Antimicrobial susceptibility pattern of the ESBL-E isolates.

As shown in Figure 2, the majority of ESBL-producing isolates were resistant to Trimethoprim-Sulfamethoxazole (94%). Moreover, 86% were resistant to Ceftriaxone and 80% were resistant to Gentamicin. The other antibiotics, with their resistance proportions, were as follows: Doxycycline (76%), Amoxicillin (72%), Ciprofloxacin (66%), Nitrofurantoin (34%), Cefepime (26%) and Amikacin (12%). Besides these, all ESBL-E isolates were susceptible to Imipenem and only 4% of these isolates were resistant to Meropenem.

All ESBL-producing strains were resistant to Trimethoprim-Sulfamethoxazole except for 2% of *E. coli.* About 87% of *E. coli* were resistant to Ceftriaxone and 75% of *Klebsiella* spp. were resistant to Amoxicillin, Ceftriaxone, Ciprofloxacin, Doxycycline and Nitrofurantoin. We found that 67% of both *Acinetobacter* spp. and *Yersinia* spp. were resistant to Ciprofloxacin, Doxycycline and Gentamicin. All *Acinetobacter* spp. and *Serratia* spp. were resistant to Ceftriaxone and Gentamicin, respectively. We observed the susceptibility of all *Acinetobacter* spp., *Enterobacter* spp., *Serratia* spp. and *Yersinia* spp. to Amikacin (Table 3).

### 3.4. Univariate Analysis

A summary of the crude logistic regression model performed to establish the relationship between the patients’ characteristics and the carriage of ESBL-E in community patients is presented in Table 4. Neither the demographic nor the clinical characteristics of the participants showed a degree of association with the carriage of ESBL-E.

## 4. Discussion

Despite the rise in the prevalence of extended-spectrum β-lactamase-producing Enterobacterales worldwide, very few reports of community ESBL-E are available from Africa. It is common in developing countries including Malawi that detection of ESBL-E has only been carried out from hospital settings, and rarely from community settings [10,12,18,19,20].

In this study, we demonstrated a low prevalence of ESBL-E isolates with high antimicrobial resistance in community settings in Blantyre, Malawi. Examining the status of ESB-producing Enterobacterales’ burden in community settings and the antimicrobial susceptibility patterns could provide important information that is a requisite for the formulation and implementation of strong infection and antimicrobial use-control strategies in both community and hospital settings.

The low prevalence found in the present study is similar to previous findings of low-to-moderate prevalence of ESBL-E in community settings in the Central African Republic [21] and Kenya [22,23]. Contrary to the higher prevalence of ESBL-E that was previously reported in a hospital setting in Blantyre [12], the low prevalence observed in this study may be an indication that community-acquired ESBL-E is still a minor problem in Blantyre, Malawi. Elsewhere, it has been reported that higher rates of antibiotic consumption accelerate both AMR selection and the increase in the rate of nosocomial ESBLs in hospital settings compared to community settings [24,25].

We found a preponderance of ESBL-E among males compared to females, but there was no statistically significant difference between ESBL-E carriage in males and females. This finding is in conformity with findings of the study by Shah et al. [26], which reported ESBL-positive isolates mostly in males (65.33%) compared to females (34.67%) in an attempt to relate age and gender to extended-spectrum β-lactamases in Enterobacterales. Another similar result was obtained in the Bhopal region of Central India where 52.54% of ESBL isolates were recovered from males compared to 43.46% from females [27]. However, our findings from the community differ from the findings of most studies conducted in hospital settings [12,28,29,30]. It has been reported that urinary tract infections mostly affect women, and samples of urine are the common sources of bacteria isolates in most microbiology laboratories in different countries [31], so whether or not the sample type (rectal vs. urine) is associated with male/female differences in ESBL-E carriage is worth future study.

In the current study, no patients with ESBL-E carriage had a history of hospital admission, but there was a higher prevalence of ESBL-E in community patients who lacked a history of surgery or antibiotic use in the past three months. These findings indicate that there was community acquisition of ESBL-E in Blantyre, Malawi.

Similar to studies that reported *E. coli* as the most frequent Enterobacterales isolate in Ethiopia [32], Burkina Faso [33], the Netherlands [34], Uganda [35] and India [27,36], the current study showed that *Escherichia coli* was the most abundant Enterobacterales species harboring extended-spectrum β-lactamase, followed by *Klebsiella* spp. in community patients in Blantyre. Probably, the reasons for *E. coli* contributing more than 60% of all isolates in the current study are the community setting and the type of sample taken. In our study, rectal samples constituted a greater number of clinical samples than urine, and *E. coli* has mostly been isolated from fecal matter versus urine [37]. Furthermore, contrary to the current study where participants with ESBL-E carriage were adult community patients who did not report a history of hospital admission in the previous three months, another Enterobacterales species, *Klebsiella* spp., has been found to cause substantial morbidity among pediatric patients (accounting for almost more than 50% of all gram-negative infections in neonates) and a significant burden when researching hospital-acquired infections in sub-Saharan Africa [31].

In the current study, we found that all ESBL-E isolates were resistant to Trimethoprim-Sulfamethoxazole except for *E. coli.* Resistance was high to Ceftriaxone, Gentamicin, Doxycycline, Amoxicillin and Ciprofloxacin. Previous studies have demonstrated a high resistance rate of ESBL-E to these first- and second-line drugs, which are always used in the management of infections caused by gram-negative bacteria [38]. High rates of resistance to the same drugs were previously observed in ESBL Enterobacterales isolated from food handlers in the Gambia [39]. Previously, it was suggested that the genetic environment necessitated the acquisition, persistence and dissemination of AMR genes in Malawi [10]. We found that Imipenem was 100% active against all isolates and only 4% were resistant to Meropenem, a group of Carbapenem antibiotics that are the choice of treatment for serious infections caused by ESBL pathogens. In fact, these drugs are not readily available for use in Malawi; therefore, limited usage may have contributed to the low resistance found in ESBL-E pathogens in the area. Similar to our results, high activity rates of Imipenem and Meropenem to ESBL-producing gram-negative bacteria were found in previous studies; all MDR *E. coli* from slaughterhouse workers in Nigeria were susceptible to both Imipenem and Meropenem [40]. Furthermore, none of the ESBL-E isolates were found to be resistant to either Imipenem or Meropenem in community settings in Cameroon [41].

In the past, studies have highlighted the risk factors for the introduction of ESBL-E into the community, including travel to areas with a higher prevalence of ESBL pathogens, previous hospitalization, antibiotic treatments, old age, comorbidities like diabetes and previous infection by members of Enterobacterales [42,43,44]. However, our study found that there was no relationship between ESBL-E carriage in community patients and their demographic or clinical characteristics. These findings are supported by the comparable results of the previous study conducted by Sanneh et al., which found that most demographic characteristics had no strong association with the carriage of ESBL-E [39]. The explanation for the lack of association between ESBL-E carriage and the demographic or clinical characteristics of patients in this study could be that all patients with ESBL-E carriage in the current study had no history of hospital admission and few had a history of prior antibiotic use in the past three months. Hospital admission and history of prior surgery have been described as the major predictors of ESBL-E carriage [45,46,47].

## 5. Limitations of the Study

We were not able to perform genotypic characterization to determine the presence of ESBL genes in Enterobacterales recovered from community patients. In addition, because Enterobacteria are usually found in the large bowel, they can easily contaminate the urinary tract, leading to contamination of the urine samples in all cases associated with fecal incontinence and/or neurological disorders impairing urinary continence. Yet, the methods used to screen and confirm ESBL production in this study had greater discriminatory power in preventing false positives, which mostly occur with the classical testing methods.

## 6. Conclusions

Our findings reveal a low presence of ESBL-E phenotypes in community patients and high rates of AMR among ESBL-E isolates. Participants with an ESBL-E carriage lacked a history of hospital admission three months before the study. This could suggest community acquisition of ESBL-E in Blantyre, Malawi, and should these carrier patients in the community remain untreated, they may serve as a community reservoir of resistant pathogen potential for the transmission and spread of community-acquired ESBLs. The low prevalence of ESBL-E in community settings in Blantyre, Malawi observed in this study can be maintained if strong infection and antimicrobial use-control strategies are implemented.

## Figures and Tables

**Table 1:** Characteristics of study population by ESBL-E phenotype.

Variables	Frequency (%)	ESBL Phenotype		*p*-Value
		Positive, n (%)	Negative, n (%)	
**Age, years**				0.63
18–27	125 (41.67)	20 (6.67)	105 (35.00)	
28–37	95 (31.67)	13 (4.34)	82 (27.33)	
38–47	43 (14.33)	10 (3.33)	33 (11.00)	
48–57	20 (6.67)	3 (1.00)	17 (5.67)	
≥58	17 (5.67)	4 (1.33)	13 (4.34)	
**Sex**				0.11
Male	137 (45.67)	28 (9.33)	109 (36.33)	
Female	163 (54.33)	22 (7.34)	141 (47.00)	
**Marital status**				0.05
Separated, divorced or widowed	23 (7.67)	8 (2.67)	15 (5.00)	
Single (never married)	93 (31.00)	14 (4.67)	79 (26.33)	
Married or cohabiting	184 (61.33)	28 (9.33)	156 (52.00)	
**Education**				0.54
Primary	133 (44.33)	23 (7.67)	110 (36.67)	
Secondary	115 (38.33)	16 (5.33)	99 (33.00)	
College/University	6 (2.00)	2 (0.67)	4 (1.33)	
Did not attend any school	46 (15.33)	9 (3.00)	37 (12.33)	
**Occupation**				0.29
Unemployed	138 (46.00)	24 (8.00)	114 (38.01)	
Self-employed or business	57 (19.00)	12 (4.00)	45 (15.00)	
Employed	78 (26.00)	8 (2.67)	70 (23.33)	
Student	27 (9.00)	6 (2.00)	21 (7.00)	
**History of prior antibiotic use in the previous three months**				0.71
Yes	66 (22.00)	10 (3.33)	56 (18.67)	
No	234 (78.00)	40 (13.33)	194 (64.67)	
**History of surgery in the previous three months**				0.54
Yes	29 (9.67)	6 (2.00)	23 (7.67)	
No	271 (90.33)	44 (14.67)	227 (75.66)	
**History of hospital admission in the previous three months**				0.23
Yes	7 (2.33)	0 (0)	7 (2.33)	
No	293 (97.67)	50 (16.67)	243 (81.00)	
**Outpatient health center**				0.20
Limbe	99 (33.00)	13 (4.33)	86 (28.67)	
Ndirande	100 (33.33)	22 (7.33)	78 (26.00)	
Zingwangwa	101 (33.67)	15 (5.00)	86 (28.67)	

**Table 2:** Composition of ESBL-E isolates from clinical samples (N = 50).

ESBL Enterobacterales	Rectal Swab	Urine	Total n (%)
*Acinetobacter baumannii*	1	0	1 (2)
*Acinetobacter lwoffii*	2	0	2 (4)
*Enterobacter aerogens*	1	0	1 (2)
*Enterobacter agglomerans*	1	1	2 (4)
*Escherichia coli*	29	4	33 (66)
*Klebsiella oxytoca*	2	0	2 (4)
*Klebsiella pneumoniae*	2	0	2 (4)
*Providencia rettgeri*	0	1	1 (2)
*Serratia liquefaciens*	0	1	1 (2)
*Serratia rubidaea*	0	1	1 (2)
*Shigella sonnei*	1	0	1 (2)
*Yersinia enterocolitica*	3	0	3 (6)
**Total, n (%)**	42 (84)	8 (16)	50 (100)

**Table 3:** Antimicrobial resistance profile of common ESBL-E isolates.

Antimicrobial Agents	ESBL-Enterobacterales (% Resistant)					
	***Acinetobacter* spp.**	***Enterobacter* spp.**	***E.coli***	***Klebsiella* spp.**	***Serratia* spp.**	***Yersinia* spp.**
Amikacin	0	0	15	25	0	0
Amoxicillin	67	67	79	75	50	33
Cefepime	33	67	21	50	0	33
Ceftriaxone	100	67	87	75	50	100
Ciprofloxacin	67	33	70	75	50	67
Doxycycline	67	67	82	75	50	67
Gentamicin	67	67	85	50	100	67
Imipenem	0	0	0	0	0	0
Meropenem	33	0	0	0	0	33
Nitrofurantoin	67	0	27	75	0	100
Trimethoprim-Sulfamethoxazole	100	100	91	100	100	100

**Table 4:** Univariate logistic regression analysis of factors associated with carriage of ESBL-E in community patients.

Associated Factor	Odds Ratio (95% CI)	*p*-Value
Age, years		
18–27	0.62 (0.18–2.09)	0.44
28–37	0.52 (0.15–1.82)	0.30
38–47	0.98 (0.26–3.71)	0.98
48–57	0.57 (0.12–3.02)	0.51
**Sex**		
Male	1.65 (0.89–3.04)	0.11
**Marital status**		
Married or cohabiting	0.77 (0.41–1.42)	0.34
**Education**		
Primary	0.86 (0.36–2.02)	0.71
Secondary	0.66 (0.27–1.63)	0.37
College/University	2.06 (0.32–13.03)	0.45
**Occupation**		
Unemployed	0.72 (0.12–1.28)	0.12
Self-employed or business	0.93 (0.31–2.83)	0.90
Employed	0.4 (0.12–1.28)	0.12
**History of surgery in the previous three months (Yes)**	1.35 (0.52–3.49)	0.54
**History of antibiotic use in the last three months (Yes)**	0.87 (0.41–1.84)	0.71

## Data Availability

The dataset used and/or analyzed in the current study is available from the corresponding author on a reasonable request.
